# Lemon Peel Polyphenol Extract Reduces Interleukin-6-Induced Cell Migration, Invasiveness, and Matrix Metalloproteinase-9/2 Expression in Human Gastric Adenocarcinoma MKN-28 and AGS Cell Lines

**DOI:** 10.3390/biom9120833

**Published:** 2019-12-05

**Authors:** Valentina Pagliara, Rosarita Nasso, Paola Di Donato, Ilaria Finore, Annarita Poli, Mariorosario Masullo, Rosaria Arcone

**Affiliations:** 1 Dipartimento di Scienze Motorie e del Benessere, Università degli Studi di Napoli “Parthenope”, Via Medina, 40, 80133 Napoli, Italy; valentina.pagliara83@gmail.com (V.P.); rosaritanasso@gmail.com (R.N.); 2 Dipartimento di Scienze e Tecnologie, Università degli Studi di Napoli “Parthenope”, Centro Direzionale Isola C4, 80143 Napoli, Italy; pdidonato@uniparthenope.it; 3 Istituto di Chimica Biomolecolare, Consiglio Nazionale delle Ricerche, Via Campi Flegrei 34, 80078 Pozzuoli (NA), Italy; ilaria.finore@icb.cnr.it (I.F.); apoli@icb.cnr.it (A.P.); 4 CEINGE, Biotecnologie Avanzate, S.C. a R.L., Via G., Salvatore, 486-80145 Napoli, Italy

**Keywords:** lemon peel polyphenols, interleukin-6, matrix metalloproteinase-9, matrix metalloproteinase-2, gastric cancer cells, cell migration, cell invasiveness, acetylcholinesterase

## Abstract

Among plant polyphenols, lemon peels extract (LPE) from the residues of the industrial processing of lemon (*Citrus limon*) shows anti-proliferative properties in cancer cells and anticholinesterase activity. In this study, we analyze the anti-cancer properties of LPE on migration and invasiveness in MKN-28 and AGS human gastric cancer cell lines either in the absence or presence of the pro-inflammatory cytokine IL-6. We find that the pretreatment with non-cytotoxic concentrations (0.5–1 μg/ml of gallic acid equivalent) of LPE inhibits interleukin-6 (IL-6)-induced cell migration and invasiveness in MKN-28 and AGS cells, as analyzed by wound and matrigel assays. Pretreatment with LPE is able to prevent either IL-6-induced matrix metalloproteinases (MMP)-9/2 activity, as assessed by gel zymography, or mRNA and protein MMP-9/2 expression, as evaluated by qPCR and Western blotting analysis, respectively. These LPE effects are associated with an IL-6-dependent STAT3 signaling pathway in MKN-28 and AGS cells. Furthermore, LPE shows acetylcholinesterase inhibitory activity when assayed by the Ellman method. In conclusion, our results demonstrate that LPE reduces the invasiveness of gastric MKN-28 and AGS cancer cells through the reduction of IL-6-induced MMP-9/2 up-regulation. Therefore, these data suggest that LPE exerts a protective role against the metastatic process in gastric cancer.

## 1. Introduction

Gastric cancer (GC) is one of the most common malignant tumors in the world, with relatively high mortality [1]. Due to the high metastatic ability of gastric cancer cells, the GC prognosis is complex to determine. A key step in the complex processes of invasion and migration of cancer cells is caused by the degradation of the extracellular matrix (ECM). Among different enzymes acting in the ECM degradation, matrix metalloproteinases (MMPs) are responsible for the collagen and protein degradation in the ECM [2,3]. MMPs are a family of zinc-dependent endo-peptidases secreted as inactive zymogens by stromal and tumor cells, and the cleavage of the pro-domain peptide yields their active form [4]. Some members of the MMP family, especially MMP-2 and MMP-9, known as gelatinases A and B, are critically involved in tumor invasion, and metastasis and their expression have been associated with poor overall survival in patients with gastric cancer [5,6,7].

Metalloproteinases level also increases during inflammation, and their secretion is promoted by pro-inflammatory cytokines. Inflammatory stimuli such as TNF-α and LPS are also implicated in gastric cancer insurgency and the metastatic process through the over-production of MMPs [1,8]. Interleukin-6 (IL-6), a pleiotropic cytokine and major mediator of inflammation, is involved in many biological processes, including cancer and autoimmune diseases [9]. This cytokine is also involved in cancer invasiveness and lymph node and/or hepatic metastasis, and it could be used as a prognostic factor for survival [10]. In fact, high levels of IL-6 have been detected in the serum of patients with gastric cancer, and the increase of IL-6 levels have been considered a predictor of poor prognosis and correlated to tumor aggressiveness [1,11]

Furthermore, increased IL-6 stimulates the activation of JAK/STAT3 signaling, functioning as transcriptional activators of STAT3 mediated target genes, which results in tumor proliferation, and/or survival. STAT3 also induces the expression of factors that promote angiogenesis, invasiveness, and/or metastasis, such as MMPs [12,13]. In particular, IL-6 exerts a pro-metastatic effect in gastric SGC7901 cancer cells that show high levels of both MMP-2 and MMP-9 in conditioned medium [1]. In addition, IL-6 significantly enhances AGS and MKN-28 gastric cell invasion [14,15].

Polyphenols from fruits and vegetables are among the major components of the health-promoting for their antioxidant properties and also for their anti-inflammatory and anti-cancer agents [16,17]. Among plant-derived polyphenols, the lemon peel extract (LPE) from the residues of the industrial processing of lemon (*Citrus limon*) has been recently studied for its anti-cancer properties in various cancer types [18].

The aim of this study is to investigate novel biological properties of LPE, in particular as an anti-cancer agent in human gastric cancer cells, and to characterize its anti-cholinesterase activity. We analyzed the protective effect of LPE on IL-6-induced migration/invasiveness or MMP-9/2 up-regulation in MKN-28 and AGS gastric cancer cells. The effects of LPE on IL-6 induced JAK2/STAT3 signaling pathways have also been investigated. Further, we analyzed the ability of LPE to inhibit acetylcholinesterase activity by in vitro enzymatic assay. Our results indicate that LPE is able to inhibit recombinant IL-6 (rIL-6)-dependent migration and invasiveness associated with the up-regulation of MMP-9 and MMP-2 expression levels in human gastric carcinoma MKN-28 and AGS cell lines.

## 2. Materials and Methods

### 2.1. Materials

Dulbecco’s modified Eagle medium (DMEM), fetal bovine serum (FBS), trypsin-EDTA, and phosphate-buffered saline (PBS) pH 7.4 were obtained from Lonza (Basel, Switzerland); rIL-6 was produced inhouse as reported [19,20]. Chemiluminescent HRP substrate kit and centrifugal filter units (Amicon Ultra 10K) were obtained from Millipore (Burlington, MA). Acetylcholinesterase from *Electrophorus electricus* (AChE) and acetylthiocholine, 5’,5’-dithiobis-2-nitrobenzoic acid (DTNB) were purchased from Sigma-Aldrich.

### 2.2. Preparation of Lemon Peel Polyphenol Extract (LPE) and Determination of Total Polyphenols’ Content

Lemon (*Citrus limon*) peels, derived from vegetable waste for liquor production, were kindly supplied by Villa Massa (Piano di Sorrento, NA, Italy). The peels were frozen at −20 °C and then lyophilized into a freeze-dry system (Labconco, 12 liter console freeze dry system). The dry solid material was sequentially ground to obtain a homogenous fine powder (particle size of about 1 mm). Polyphenols were recovered by maceration as follows: 1 g of powdered waste was suspended into 25 ml of 80% EtOH and left under stirring at room temperature for 2 h. Partial purification was performed by discarding the resting solid material by centrifugation at 10,000× *g* for 40 min (Centrifuge Avanti^TM^ J-25, rotor JA14 Beckman Coulter^TM^). The liquid phase was filtered on paper-filter (Whatman® cellulose Grade 1) and successively reduced to 10–15 ml in a rotary evaporator (Buchi Rotavapor R-210) at 40 °C under vacuum. The total phenolic content in the extract was determined according to the adapted Folin–Ciocalteu colorimetric method [21] and the results were expressed as mg Gallic acid equivalent (GAE) per g of dry sample. The raw extracts were concentrated at 40 °C under vacuum by a rotary evaporator and dissolved in the suitable solvent for the biological assays.

### 2.3. Cell Cultures and Treatments

The human gastric adenocarcinoma cell lines MKN-28 [17] and AGS (American Type Culture Collection, ATTC CCL-107) were cultured in Dulbecco’s modified Eagle’s medium (DMEM) supplemented with 10% heat-inactivated fetal bovine serum (FBS) (Invitrogen, Life Technologies, Italy), 1.5 mM L-glutamine, 100 units/ml penicillin, and 100 μg/ml streptomycin under humidified atmosphere of 5% CO_2_ at 37 °C. The treatments of sub-confluent cells with increasing concentration (10–100 ng/ml) of rIL-6 [19,20] or lemon peel extract (LPE) (0.5–50 μg/ml) [18] were performed for 24 h in serum-free DMEM to determine the IC_50_ value. The evaluation of LPE protective effects was performed by the pretreatments with two concentrations of LPE (0.5 or 1 μg/ml) for 6 h that was followed by exposure to rIL-6 (50 ng/ml) for further 24 h. The treatments were performed under serum-free conditions in the presence of 0.5% (*v/v*) final concentration EtOH used as a vehicle for LPE.

### 2.4. Cell Proliferation Assay

Cell viability was evaluated as mitochondrial metabolic activity using the PrestoBlue^TM^ (PB) cell viability reagent (Cat. N. A13262, Invitrogen, Life Technologies, Italy) [22], according to the manufacturer’s protocol. Briefly, the cells were plated onto 96-well plates (1 × 10^4^ cells/well), treated and then incubated with (PB) reagent at 10% final concentration for 1 h. The absorbance was read at 570 nm with a reference wavelength set at 600 nm using a microplate reader (Labsystems Multiskan, MS). Control wells without cells, containing DMEM in the absence or the presence of the different LPE concentration, were used for background subtraction. The cell viability was expressed as a percentage relative to the untreated cells, cultured in serum-free medium, set as 100%.

### 2.5. RNA Extraction, Reverse Transcription (RT), and Quantitative Real-Time Polymerase Chain Reaction (qPCR)

Total RNA was purified by the ultrapure TRizol reagent (GibcoBRL) according to the manufacturer’s instructions. The concentration and purity of RNA were determined spectrophotometrically by reading the absorbance at 260 and 280 nm. Aliquots (1 μg) of total RNA were subjected to DNase I digestion (Thermo Fisher Scientific) and reverse transcribed using Sensi FAST^TM^cDNA synthesis kit (Cat. N. BIO-65054, Bioline, Italy) according to the manufacturer’s protocol. Real-time PCR was carried out using the PowerUP Sybr green master mix (Thermo Fisher Scientific), the Quant Studio 7 Flex instrument, and the fast gene-expression method with the following conditions: a first denaturation step a 95 °C for 20 s followed by 40 cycles at 95 °C for 1 s, 60 °C for 30 s and then melting curve analysis was performed raising temperature from 60 °C until 95 °C with a 0.5 °C/s increase. Reactions were carried out in triplicate, and the 18S gene was used as an internal control to normalize the variability in expression levels. The 2^−ΔΔCT^ (cycle threshold) method [20] was used to calculate the results and mRNA expression levels were determined as fold-induction relative to Ctrl cells, set as 1. PCR primers for MMP-2, MMP-9, and 18S were designed using Primer Blast software based on sequence deposited in the GeneBank Database (NCBI: NM_004530.5, NM_004994.2, and NR_145820.1, respectively). Reactions were carried out in triplicate, and the 18S gene was used to normalize the variability in expression levels. The mRNA expression levels were determined as fold-induction relative to control cells, set as 1. The primers used for the qPCR reactions are the following:

$$\begin{array}{l} MMP-2\;forward:\;5^{\prime }-GAACTTCCGTCTGTCCCAGG-3^{\prime }\\ \;\;\;\;\;\;\;\;\;\;\;\;\;\;\;\;\;\;\;\;\;\;\;reverse:\;5^{\prime }-AATGAACCGGTCCTTGAAGAAGA-3^{\prime }\\ MMP-9\;forward:\;5^{\prime }-TGTTCAAGGATGGGAAGTACTGG-3^{\prime }\\ \;\;\;\;\;\;\;\;\;\;\;\;\;\;\;\;\;\;\;\;\;\;reverse:\;5^{\prime }-GTGTGTACACCCACACCTGG-3^{\prime }\\ 18S\;\;\;\;\;\;\;\;\;\;\;forward:\;5^{\prime }-CGATGCGGCGGCGTTATTC-3^{\prime }\\ \;\;\;\;\;\;\;\;\;\;\;\;\;\;\;\;\;\;\;\;\;\;reverse:\;5^{\prime }-TCTGTCAATCCTGTCCGTGTCC-3^{\prime } \end{array}$$

### 2.6. Western Blotting Analysis

Cells were seeded in 6-well plates (2 × 10^5^ cells/well) and subjected to different treatments. Whole-cell protein extracts were prepared by lysing cells in 50 mM Tris-HCl pH 8.0, 150 mM NaCl, 0.5% sodium deoxycholate, 0.1% SDS, 1 mM EDTA, 1% Igepal, 1 × protease inhibitor (cat. n. 11836153001, Roche Applied Science, Italy), and a phosphatase inhibitor cocktail (cat. n. 524627, Calbiochem, Italy). Protein concentration was determined by a colorimetric assay [23]. The culture medium was harvested and contaminating cells and debris were removed by centrifugation at 6000× *g* for 20 min at 4 °C. The cell supernatant was then concentrated approximately 20-fold by centrifugation at 5000× *g* using Ultra-4, PLGC Ultracell-PL Membrane, 10 kDa cut-off (cat. n. UFC 801024, Merck Millipore, Italy) at 4 °C. Whole-cell protein extracts (40 μg) and appropriate volumes of concentrated conditioned media, (corresponding to 30 μg total cell proteins) were heated at 95 °C for 5 min in Laemmli denaturing buffer in the presence of 2 M urea and then loaded onto 12% reducing SDS-PAGE [24]. After electrophoresis, proteins were transferred to a PVDF membrane (GE Healthcare Life Sciences, Italy) that was incubated with a Ponceau–S solution for protein staining; the membrane was then exposed to the primary antibody. The following antibodies were used: rabbit polyclonal antibody raised against phospho-STAT3 (Ser 727) (1:1000, Cat. N. 9134, Cell Signaling Technology, Euroclone, Italy); STAT3 (1:1000, Cat. N. 9139, Cell Signaling Technology, Euroclone, Italy); glyceraldehyde-3-phosphate dehydrogenase (GAPDH) antibody (Cat. N. AM4300, Ambion, Applied Biosystems, Italy) was used as protein loading control. After incubation with the appropriate peroxidase-linked secondary antibody, an Immobilon Western chemiluminescent HRP substrate (ECL) kit (Cat. No. WBKLS0500, Millipore Corporation, USA) was used for visualization. Densitometric analysis of the ECL signal was performed using the free image-processing software ImageJ, version 1.47 (https://imagej.nih.gov/ij/download.html).

### 2.7. Gelatin Zymography

Gelatinolytic activity in the cell-conditioned medium was assayed by SDS-PAGE zymography, as described previously [7,17]. Samples were analyzed under non-reducing conditions without boiling, through a 12% SDS-polyacrylamide gel co-polymerized in the presence of gelatin (1 mg/ml, cat. n. G1890, Sigma-Aldrich, Italy). Electrophoresis was conducted at 35 mA for 90–120 min at 4 °C. After the run, the proteins in the gels were renatured in a 2.5% Triton X-100 solution for 1 h. The gels were then incubated with 50 mM Tris-HCl pH 7.5, 200 mM NaCl, 5 mM CaCl_2,_ and 5 μM ZnCl_2_ at 37 °C for 48 h, which allows substrate degradation. Finally, the gels were fixed in 30% methanol, 10% acetic acid for 30 min, and stained with 0.5% Coomassie Brillant Blue R-250. Proteolytic bands were visualized by destaining with 50% methanol and 5% acetic acid.

### 2.8. Wound Assay

The cell migration was assessed using a wound assay [25]. Cells (2 × 10^5^ cells/well) were seeded into each well of a 6-well plate and incubated with complete medium at 37 °C and 5% CO_2_. After 24 h of incubation, the cells were scrapped horizontally and vertically with a sterilized P10 pipette tip, subjected to different treatments in medium with 0.5% FBS and two views on the cross were photographed on each well at 0, 12, and 24 h, using a Zeiss Axiovert 40 CFL inverted microscope (Carl Zeiss, Milan, Italy) 10 × objective. The microscope was equipped with a 12.1-megapixel CCD digital video camera (Canon, PowerShot G9, Italy) with a digital image software (Remote Capture DC, Canon). Quantitative analysis of the scratch assay was performed by measuring the gap area using the free image-processing software ImageJ version 1.47.

### 2.9. Matrigel Invasion Assay

Cell invasion assay was performed by BD Falcon BioCoat Matrigel invasion chambers (cat. n. 354480, BD Bioscience, Bedford MA; 8 μm). The cell culture inserts were rehydrated and prepared as previously described [7,17]. Briefly, 2 × 10^4^ cells in 0.5 ml of DMEM with 0.5% FBS were seeded in the upper chamber, and 750 μl medium with 5% FBS was placed in the lower chamber. After 24 h, cells in the upper chamber were removed with the cotton swab, and the cells at the bottom of the filters were fixed and stained with a Diff-Quick kit (cat. n. B4132-1A, Becton-Dickinson). After two washes with water, the inserts were allowed to air dry, and phase-contrast images were captured as described above using an LD A-Plan 20×/0.30 Ph 1 objective. To assess cell invasion ability, the total number of cells was determined by cell counting in five fields randomly selected per membrane.

### 2.10. Acetylcholinesterase Inhibition Assay

Cholinesterase activity was assayed by the Ellman method [26] using acetylthiocholine as substrate. The reduction of dithiobisnitrobenzoate by the thiocholine, produced by the enzymatic hydrolysis of thiolated substrates, was followed colorimetrically (412 nm) at room temperature (22–27 °C). Briefly, an 1 ml aliquot of LPE was dried under a liquid nitrogen stream, and then the pellet was resuspended in the same volume of PBS. The reaction mixture (500 µL) contained 330 µM 5,5’-dithio-bis-2-nitrobenzoic acid (DTNB), 500 µM acetylthiocholine as substrate, and different amounts of LPE (7–60 µg/ml final concentration) in 0.1 M sodium phosphate buffer pH 7.4. The reaction was started by the addition of 100 mU/ml AChE, and the initial rate of the reaction was derived from the linear portion of the kinetics. The concentration of LPE required to reduce the enzymatic activity to 50% (IC_50_) was derived from semi-logarithmic plots. Linear curve fits were obtained with the least-squares method, and the significance of the correlation was estimated from the squared correlation coefficient r^2^.

### 2.11. Statistical Analysis

The data were expressed as mean ± SD of at least three independent experiments performed in triplicate. Statistical significance of treated samples against control cells (cultured in serum-free medium) was determined by a one-way analysis of variance (ANOVA), followed by Bonferroni’s test (*p*-values of * *p* < 0.05; ^§^ *p* < 0.01; ^#^ *p* < 0.001).

## 3. Results

### 3.1. Effect of LPE Treatment on Cell Proliferation

**Figure 1:**
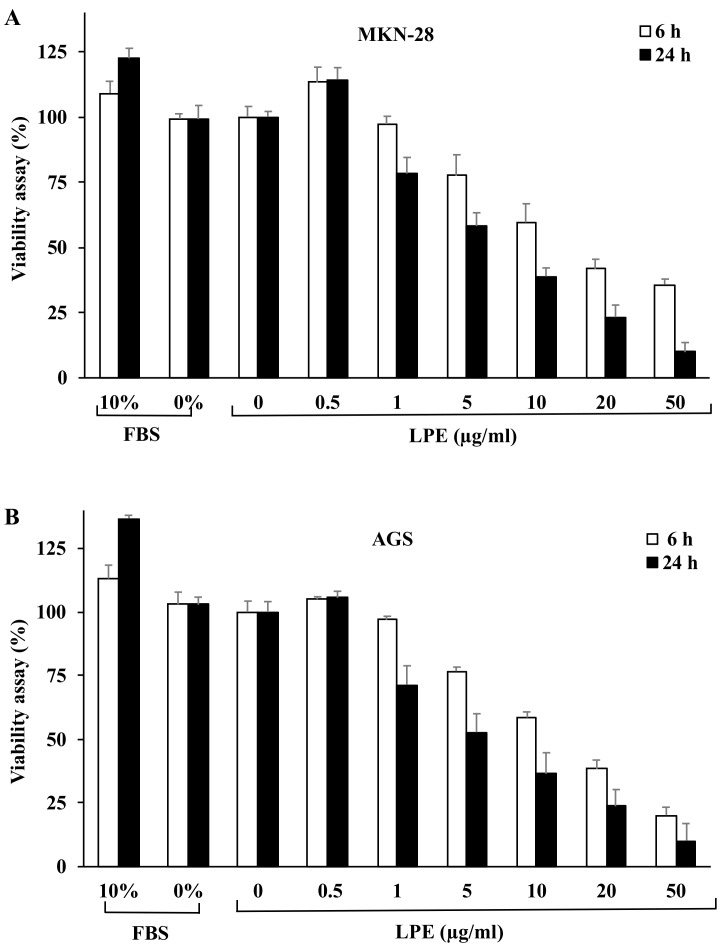
Effect of LPE treatment on MKN-28 and AGS cell proliferation. Cells exposed to increasing concentrations of LPE (0.5–50 µg/ml GAE), in serum-free medium for 6 and 24 h, were subjected to cell viability assay. (**A**) Viability of MKN-28 and (**B**) AGS cells was quantitated by the cell viability reagent at indicated times of LPE exposure. Results are presented as percentage (mean ± SD) (*n* = 3) of the control cells, cultured in serum-free DMEM with 0.5% (*v*/*v*) EtOH (vehicle), set as 100%.

In a first approach, we analyzed the ability of the LPE (prepared as reported in Section 2.2) containing a total polyphenol content of 0.46 mg/ml GAE to affect proliferation of MKN-28 and AGS cells. To this aim, the cell lines were exposed to increasing amounts of LPE, ranging from 0.5 to 50.0 μg/ml [18] in serum-free conditions for 6 and 24 h and then subjected to a viability assay. As shown in Figure 1, cell proliferation was inhibited by LPE in a time and concentration-dependent manner in a range from 1.0 to 50.0 μg/ml GAE.

**Figure A1:**
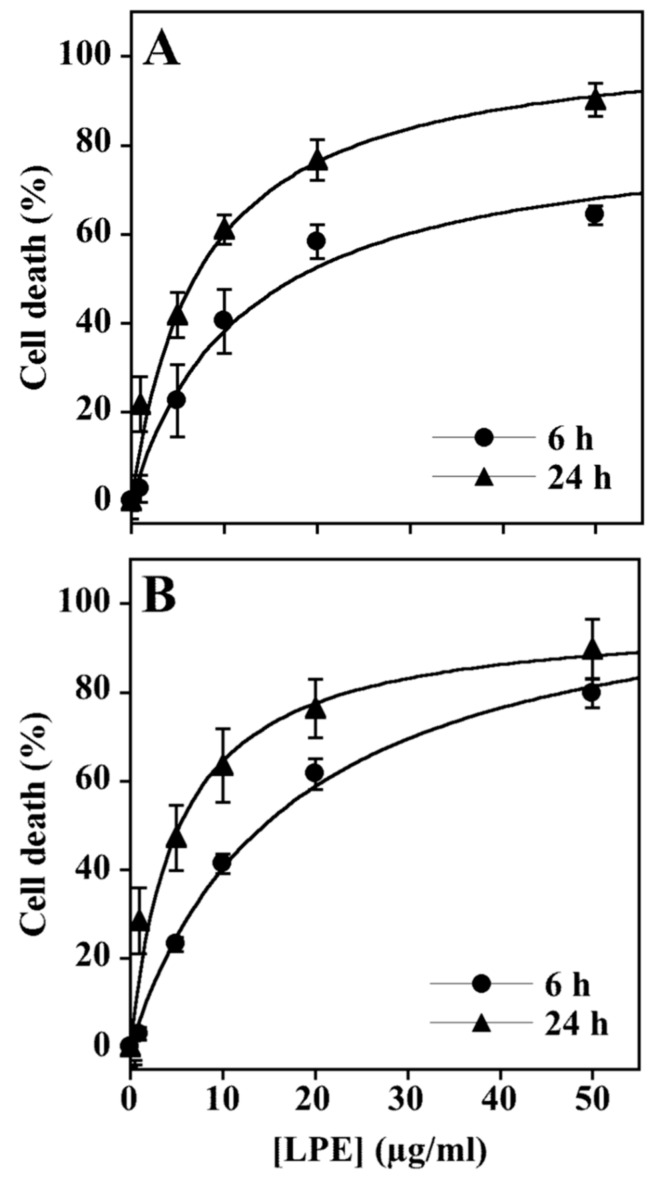
Determination of LPE IC_50_ values on (**A**) MKN-28 and (**B**) AGS cells. Cells exposed to increasing concentrations of LPE (0.5–50 µg/ml GAE), in serum-free medium for 6 and 24 h, were subjected to cell viability assay by the PrestoBlue reagent. Data were determined by a non-linear regression analysis by an hyperbolic binding equation (r^2^ > 0.968 and 0.971 for MKN-28 cells at 6 or 24 h, respectively).

The IC_50_ values expressed as GAE were determined by non-linear regression analysis in a hyperbolic binding equation (Figure A1) at 6 (12.14 and 17.06 μg/ml for MKN-28 and AGS, respectively), and 24 h stimulation (7.46 and 5.08 μg/ml for MKN-28 and AGS, respectively). In the light of these results, the two concentrations of 0.5 μg/ml, that did not affect cell viability and 1.0 μg/ml that exhibited little effect on the cell viability (~90%), were selected and used in the subsequent experiments to evaluate the protective effect of LPE on IL-6 exposed MKN-28 and AGS cells.

### 3.2. The LPE Treatment Reduces the rIL-6-Stimulated Migration and Invasiveness of MKN-28 and AGS Cells

**Figure 2:**
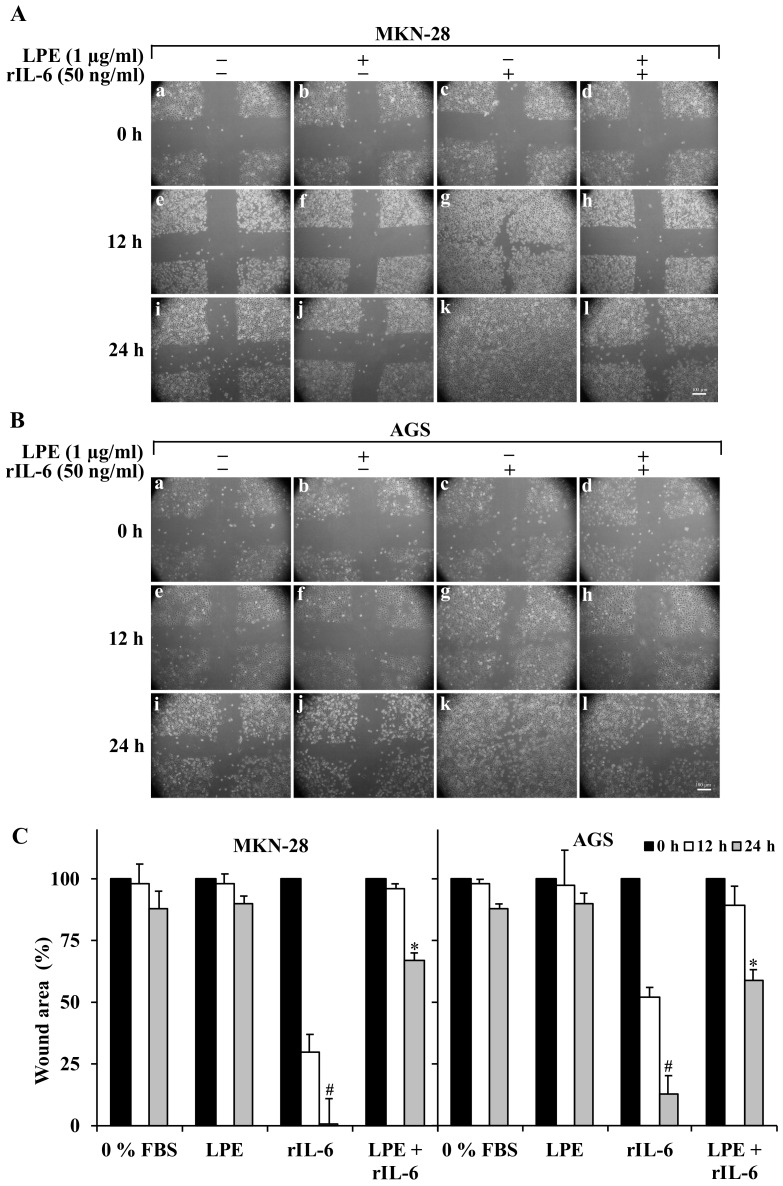
Effect of LPE exposure on rIL-6-induced cell migration of MKN-28 and AGS cells. Representative images of the wound healing assay of (**A**) MKN28 and (**B**) AGS cells captured at time 0, 12, and 24 h by a phase-contrast-microscope (10 × objective). Cells kept in serum-free DMEM (**a**–**e**,**i**) were treated with LPE alone (**f**,**j**) rIL-6 alone (**g**,**k**) or pre-treated with LPE (1 μg/ml GAE) for 6 h and then exposed to rIL-6 (50 ng/ml) for 12 h (**h**) or 24 h (**l**). (**C**) Quantitative analysis of wound area of MKN-28 and AGS cells at time 0, 12, and 24 h. For each treatment, data show the wound area at the indicated time in comparison to that of the open wound at time 0, set as 100%. Results are presented as mean ± SD (*n* = 3). (^#^ *p* < 0.001, statistically significant vs. untreated cells and * *p* < 0.05 vs. treated cells with rIL-6).

Since IL-6 causes an increase of migration and invasiveness in gastric cancer cells [1,14,15,27,28], we investigated the effect of LPE treatment on rIL-6-induced migration and invasive ability by wound healing and matrigel invasion assays, respectively, in MKN-28 and AGS cells. The wound-healing assay (Figure 2) shows that either serum deprivation (Panel A,B images a,c,i) or the treatment with LPE alone (Figure 2, Panel A,B images f,j) do not affect the wound area compared to that of untreated cells (Figure 2, Panel A,B images a,e,i) in both cell lines.

Conversely, in both cell lines, the exposure to rIL-6 alone led to a significant reduction in the wound area (Figure 2, Panel A,B images g,k) compared to that of untreated cells. Quantitative analysis of wound area (Figure 2, Panel C) shows that the effect induced by rIL-6 is more prominent in MKN-28 cells that show a major percentage of wound area reduction (~70% or 97% at 12 and 24 h, respectively) compared to AGS cells (~50% or 90%, at 12 and 24 h, respectively). Interestingly, the pre-treatment with LPE prevents the rIL-6 induced reduction of wound area, either in MKN-28 (Figure 2, Panel A images h,l) (~5% or 40% at 12 and 24 h, respectively) or in AGS (Figure 2, Panel B images h,l) (~10% or 30% at 12 and 24 h, respectively) as compared to the cells stimulated with rIL-6 alone.

**Figure 3:**
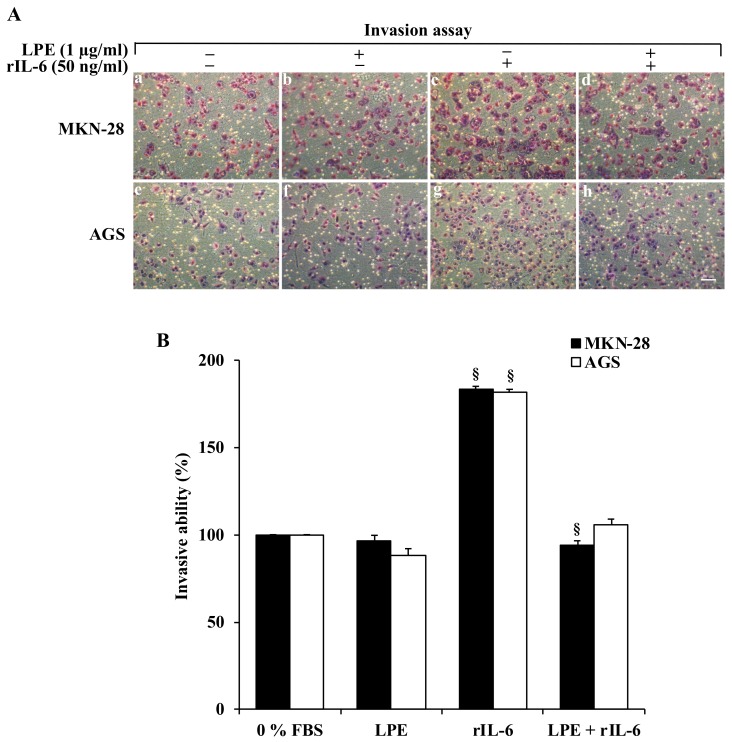
Effect of LPE treatment on rIL-6-induced cell invasion of MKN-28 and AGS cells. Cells seeded in trans-well chambers for the matrigel invasion assay were pretreated with LPE (0.5 or 1 μg/ml GAE) for 6 h and then exposed to rIL-6 (50 ng/ml) for further 24 h. Thereafter, the cells at the bottom of the filters were fixed, stained, and observed by a phase-contrast-microscope (10 × objective). (**A**) Representative photomicrographs of random fields of MKN-28 and AGS cells keep in serum-free medium (**a**,**e**); exposed to LPE alone (**b**,**f**); treated with rIL-6 alone (**c**,**g**); pretreated with LPE and then exposed to rIL-6 (**d**,**h**). (**B**) The invasive capability was determined by cell counting in five fields, randomly selected, per membrane. Quantification was relative to untreated cells, cultured in serum-free DMEM, set as 100%. Results are presented as mean ± SD (*n* = 3). (^§^ *p* < 0.01, statistically significant vs. untreated cells or treated with rIL-6).

The matrigel invasion assay (Figure 3) shows that, in both cell lines, rIL-6 induces a 2-fold increase in number of cells that had invaded through the membrane (Figure 3, Panel A images c,g) compared to untreated (Figure 3, Panel A images a,e) or LPE exposed cells (Figure 3, Panel A images b,f).

Interestingly, the pre-treatment with LPE leads to an invasive ability similar to that observed in the absence of the inflammatory stimulus caused by rIL-6 (Figure 3, Panel B).

These results clearly indicate that non-cytotoxic LPE concentrations prevent the rIL-6 exerted pro-metastatic effect in MKN-28 and AGS gastric cancer cells.

### 3.3. Effect of Exposure to rIL-6 on MMP-9 and MMP-2 Gelatinolytic Activity and Expression Levels in MKN-28 and AGS Cells

**Figure A2:**
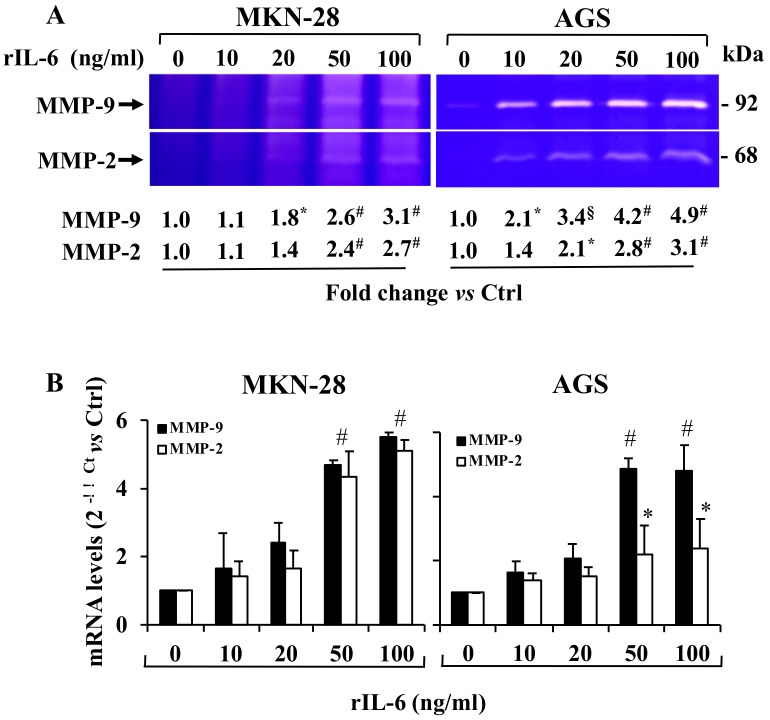
Effect of rIL-6 treatment on MMP-9 and MMP-2 gelatinolytic activity and mRNA expression levels in MKN-28 and AGS cells. (**A**) Gelatin zymography under non-reducing conditions of conditioned media from MKN-28 and AGS cells. (**B**) Quantitative qPCR analysis of MMP-9 and MMP-2 mRNA expression levels in MKN-28 and AGS cells; 18S was used as internal control. The relative fold change vs. untreated cells, set as 1, of protein levels is shown under each lane.

As cell invasiveness is strictly related to IL-6-induced upregulation of MMP-9 and MMP-2 expression levels in human gastric cancer cells [1,10,15], we have explored the effect of LPE in rIL-6 exposed MKN-28 and AGS cells. To this aim, we first analysed the effect of increasing amounts of rIL-6 (10–100 ng/ml) on MMP-9/2 gelatinolytic activity and expression levels. Gel zymography performed on cell-conditioned media from MKN-28 and AGS cells (Figure A2, Panel A) revealed that rIL-6 was able to induce MMP-9 and MMP-2 gelatinolytic activity in a concentration-dependent manner, with a maximum increase of 3.1- and 4.9-fold for MMP-9 and 2.7- and 3.1-fold for MMP-2, respectively, in MKN-28 and AGS cells compared to that of unstimulated cells. The increase of gelatinolytic activity was detected in both cell lines, although to a major extent in AGS cell-conditioned media.

We also evaluated whether this increase of MMP-9/2 gelatinolytic activity was due to an rIL-6-dependent increase of MMP-9/2 mRNA expression levels. The result of qPCR analysis (Figure A2, Panel B) shows that both MMP-9 and MMP-2 mRNA expression levels, which were very low in untreated cells, greatly increased both in MKN-28 and AGS cells exposed to rIL-6. The up-regulation of MMP-9/2 expression level was concentration-dependent in both cell lines; however, the maximal induction of MMP-9/2 mRNA expression levels was reached at 50 ng/ml rIL-6, in agreement with previous result [27]. Therefore, this concentration was chosen for further studies.

The above data indicate that rIL-6-induces an up-regulation of MMP-9 and MMP-2 gelatinolytic activity, which is correlated to an increase of their mRNA expression level in MKN-28 and AGS cells.

### 3.4. Effect of LPE on rIL-6 Induced Up-Regulation of MMP-9/2 Expression Levels in MKN-28 and AGS Cells

**Figure 4:**
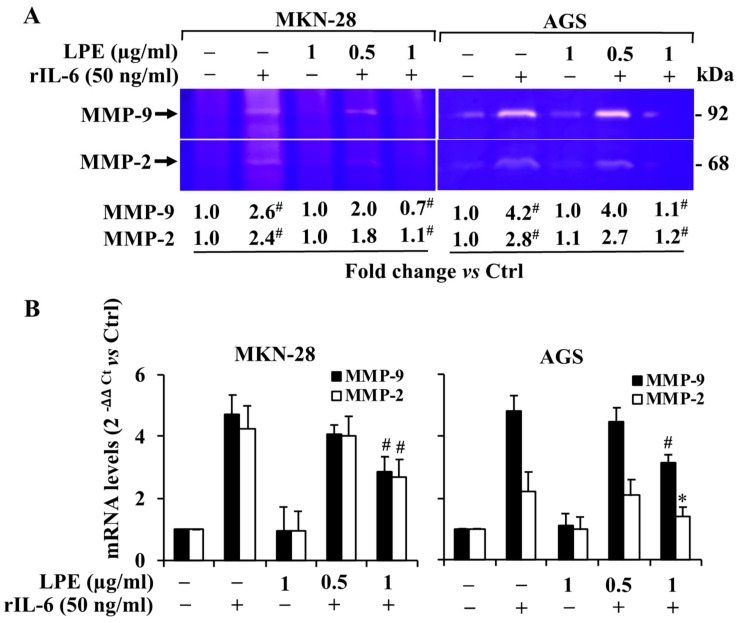
Effect of LPE exposure on rIL-6-induced MMP-9 and MMP-2 gelatinolytic activity and expression level in MKN-28 and AGS cells. Cells, pretreated with LPE (0.5 or 1 μg/ml GAE) for 6 h, were then exposed to rIL-6 (50 ng/ml) for further 24 h. After the treatments, the cells were harvested for preparation of total RNA and protein extracts. Cell conditioned media were collected, concentrated by ultrafiltration, and volumes corresponding to 40 μg of cellular proteins were analyzed under non-reducing conditions through a 12% SDS-polyacrylamide gel co-polymerized in the presence of gelatin (1 mg/ml) for (**A**) gelatin zymography of MKN-28 and AGS cells. (**B**) Quantitative qPCR analysis of MMP-2 and MMP-9 mRNA expression levels of MKN-28 and AGS cells. 18S was used as the internal control. Results are presented as mean ± SD (*n* = 3). (* *p* < 0.05; # *p* < 0.001, statistically significant from untreated cells or treated cells with rIL-6).

To analyze the effect of LPE on rIL-6-induced MMP-9/2 expression levels, the conditioned medium of cells pretreated with LPE and then exposed to rIL-6 were subjected to gelatin zymography and qPCR analysis (Figure 4). The pre-treatment with LPE reduces the rIL-6-induced MMP-9 and MMP-2 gelatinolytic activity in the culture medium of both cell lines (Figure 4, Panel A). This effect was LPE concentration-dependent, and the highest concentration tested (1 μg/ml GAE) completely suppressed the rIL-6 induced MMP-9/2 enzyme activity up-regulation, which was similar to that observed in untreated cells.

Similar behavior was observed when the effect of the LPE pre-treatment was evaluated on rIL-6 dependent induction of MMP-9/2 mRNA expression level (Figure 4, Panel B). The pre-treatment with 1 μg/ml LPE for 6 h induces a decrease of IL-6 induced MMP-9/2 mRNA expression levels in both cell lines. However, as can be observed (Figure 4, Panel B), LPE greatly reduces the effect on the MMP-2 mRNA levels that are stimulated by rIL-6 in AGS cells. In addition, the exposure to LPE is able to counteract the rIL-6 induced MMPs mRNA levels up-regulation although to a different extent in MKN-28 or AGS cells. In particular, the highest reduction in mRNA expression has been observed for MMP-2 levels in AGS cells. In the latter case, the pre-exposure to 1 µg/ml LPE leads a MMP-2 expression level that is similar to that on untreated cells.

### 3.5. Effects of LPE on IL-6-Dependent STAT3 Phosphorylation Levels in MKN-28 and AGS Cells

**Figure 5:**
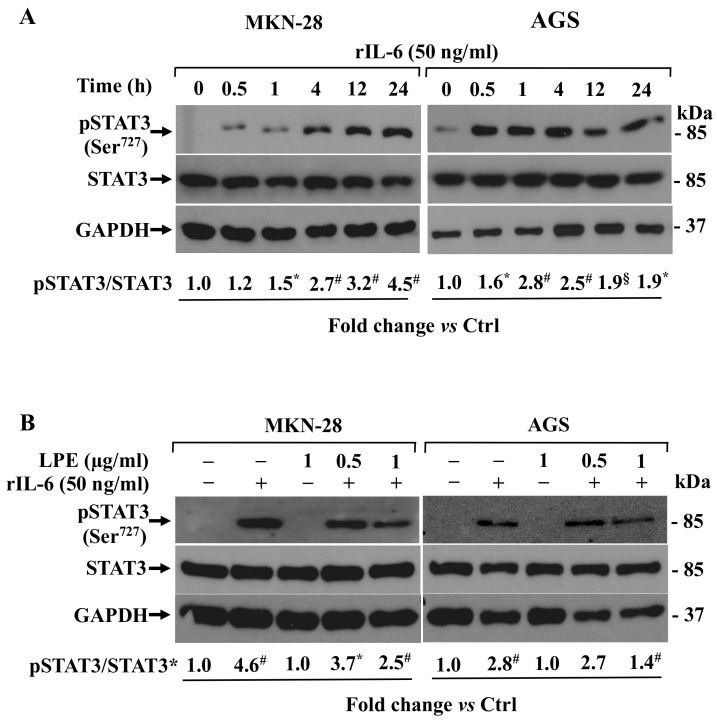
Effect of LPE exposure on rIL-6 induced on STAT3 phosphorylation level in MKN-28 and AGS cells. **(A)** Western blotting analysis of phospho-STAT3 expression levels in cells treated with rIL-6 for the indicated times or **(B)** preincubated with LPE (expressed as GAE) and then exposed to rIL-6. The relative fold change vs. untreated cells, set as 1, of protein levels is shown under each lane.

As the IL-6-induced transcriptional stimulation of MMP-9/2 results from the activation of STAT3 via the JAK2/STAT3 pathway in gastric cancer and AGS cells [27,28], we tested the STAT3 phosphorylation level [29] in MKN-28 and AGS cells exposed to rIL-6 for different time points. Panel A in Figure 5 shows that in untreated cells, kept in serum-free medium, a negligible amount of pSTAT3 protein level is detectable whereas, remarkably, the treatment with IL-6 strongly stimulated STAT3 phosphorylation at Serine 727 residue (Ser^727^STAT3) in a time-dependent manner.

The pSTAT3 protein level progressively increased until 24 h in MKN-28 cells (~4.5-fold); conversely, in AGS cells, a ~1.9-fold increase was observed at 12 h stimulation. In both cell lines, no difference in STAT3 total protein level was observed in comparison to pSTAT3 levels. The pretreatment with LPE (Figure 5, Panel B) was able to reduce the rIL-6 stimulated pSTAT3 level compared to that of cells exposed to rIL-6 alone, although with a different efficacy between the two cell lines. The major inhibitory effect was observed at the higher LPE concentration (1 µg/ml GAE) that yield ~2-fold decrease of pSTAT3/STAT3 level compared with that observed in cells treated with rIL-6 alone, in both cell lines. A low pSTAT3 signal was immuno-detected in cells treated with LPE alone that was similar to that of unstimulated cells, and no changes in the abundance of the total STAT3 protein level were found in the cells exposed to the different treatments. These findings indicate that LPE can affect the IL-6-dependent STAT3 phosphorylation in MKN-28 and AGS cells.

### 3.6. Effect of LPE on Acetylcholinesterase Activity

**Figure 6:**
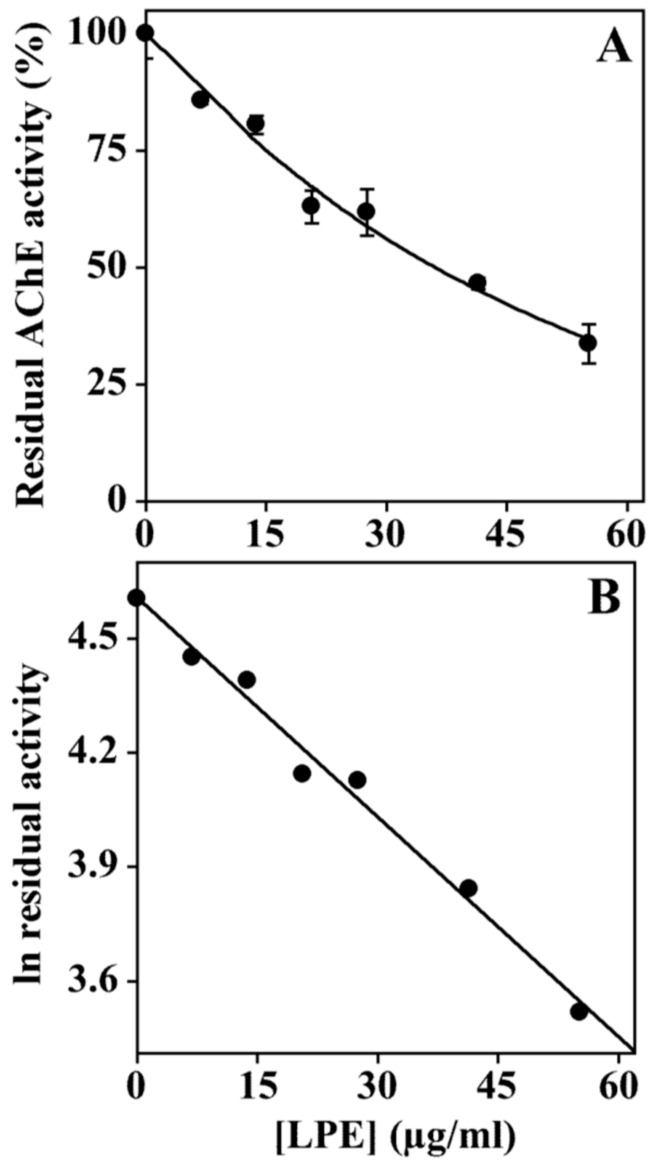
Effect of LPE on AChE activity. (**A**) AChE activity was assayed in the absence or the presence of the indicated amounts of LPE (expressed as GAE). (**B**) Data were analyzed according to a first-order behaviour, and IC_50_. was calculated from the slope of the linear regression (r^2^ = 0.995).

Up to today, acetylcholinesterase inhibitors represent the major approved drugs to treat Alzheimer’s disease, and therefore, many efforts have been devoted to the identification of novel compounds able to inhibit AChE as potential drugs for the treatment of this neurodegenerative disease [30,31]. Since a previous study [18] demonstrated that LPE shows an acetylcholinesterase inhibitory activity when assayed by TLC bioautographic assay [32], we performed a further characterization of this activity. We used an in vitro AChE enzyme assay [26] that allows an accurate determination of the inhibition values. The assay was performed in the absence or the presence of different LPE concentrations, and the result (Figure 6) indicates that LPE exhibits AChE inhibitory activity (Figure 6).

The analysis of the data by a semilogarithmic plot (Figure 6, Panel B) allowed the determination of the inhibitor concentration required to get inhibition of half of the activity (IC_50_) corresponding to 36 µg/ml GAE, a result in agreement with those previously obtained [18].

## 4. Discussion

Several studies on dietary polyphenols have demonstrated their chemopreventive activities [17,33,34,35]. Regarding lemon polyphenols, the exploitation of their different beneficial effects on human health, such as the reduction of blood glucose levels, the regulation of lipid metabolism, and the reduction of inflammation, has already been reported [36,37,38]. In previous studies, the lemon peel industry’s wastes have been reported to represent a sustainable source of such valuable phenolic compounds [18,39,40]. HPLC analysis of these residues’ extracts showed the presence of main phenolic compounds such as benzoic and *p*-coumaric acids, hesperetin, naringenin, naringin, and quercetin [18]. This polyphenolic mixture exhibited promising properties as antioxidant and anti-proliferative agents against some cancer cells [18]. These results prompted us to further investigate the biological potential of lemon wastes’ polyphenols as anticancer agents.

The results here reported demonstrate novel biological properties of LPE that are able to prevent IL-6-induced invasiveness, to reduced IL-6-stimulated MMP-9/2 up-regulation in gastric MKN-28 and AGS cancer cell lines, and to inhibit in vitro AChE activity. First, we demonstrated that LPE affects cell proliferation in a concentration-dependent manner in MKN-28 and AGS cells in agreement with the anti-proliferative effect observed in neuroblastoma SH-SY5Y, colorectal adenocarcinoma Caco-2, and hepato-carcinoma HepG2 human cells (1 μg/ml GAE) [18].

As the inflammatory cytokine IL-6 also promotes migration and invasiveness of gastric cancer cells [15], we analyzed the effect of LPE in the IL-6 stimulated human gastric AGS and MKN-28 cell lines. We observed that the pre-treatment with LPE is able to counteract the rIL-6-induced increase in the migration and invasive abilities of MKN-28 and AGS cells, in agreement with the scientific literature [1,14,15,27,28].

Here we also demonstrated that LPE is able to prevent the rIL-6-induced up-regulation of MMP-9 and MMP-2 gelatinolytic activity in the conditioned medium of MKN-28 and AGS cells. Further, we demonstrated that LPE chemo-preventive effects are correlated with MMP-9 and MMP-2 down-regulation both at mRNA and protein expression levels.

Following the IL-6 binding to its transmembrane receptor gp130, the activation signal transduction requires the activation of Janus kinase (JAK), which is followed by phosphorylation of STAT (STAT1 and STAT3) [20], thus, suggesting that JAK-STAT pathways play an important role in the gastric cancer process. We found that rIL-6 increased the STAT3 serine phosphorylation both in MKN-28 and AGS and this effect was inhibited by LPE pre-exposure before rIL-6 stimulation. This data strongly demonstrates that LPE is able to prevent rIL-6 induced effects by impairing the JAK2/STAT3 activation pathway in MKN-28 and AGS cells.

## 5. Conclusions

In conclusion, our results demonstrate that LPE appears to be a protective agent against the insurgence of human gastric adenocarcinoma since it can reduce the upregulation of key enzymes such as MMP-9 and MMP-2 that occur during the inflammation process. Moreover, LPE also shows a neuroprotective effect for its ability to inhibit AChE activity, an enzyme involved in Alzheimer’s disease. Although further studies will be necessary to clarify the molecular and cellular mechanisms underlying LPE anticancer effects, our data highlight the beneficial effects of LPE in the prevention and/or therapy of human gastric cancer metastasis and Alzheimer’s disease. Moreover, the biological properties of LPE could be exploited not only in the pharmaceutical field but also in food industries. In fact, LPE preparation represents a useful strategy for the recycling of vegetable waste that allows the production of novel additives or functional compounds with new biological properties.
